# Structural and Fluorine Plasma Etching Behavior of Sputter-Deposition Yttrium Fluoride Film

**DOI:** 10.3390/nano8110936

**Published:** 2018-11-14

**Authors:** Wei-Kai Wang, Yu-Xiu Lin, Yi-Jie Xu

**Affiliations:** Department of Materials Science and Engineering, Da-Yeh University, Changhua 51591, Taiwan; h63290@gmail.com (Y.-X.L.); pa821221@yahoo.com.tw (Y.-J.X.)

**Keywords:** yttrium fluoride, films, plasma processing equipment

## Abstract

Yttrium fluoride (YF_3_) films were grown on sapphire substrate by a radio frequency magnetron using a commercial ceramic target in a vacuum chamber. The structure, composition, and plasma etching behavior of the films were systematically investigated. The YF_3_ film was deposited at a working pressure of 5 mTorr and an RF power of 150 W. The substrate-heating temperature was increased from 400 to 700 °C in increments of 100 °C. High-resolution transmission electron microscopy (HRTEM) and X-ray diffraction results confirmed an orthorhombic YF_3_ structure was obtained at a substrate temperature of 700 °C for 2 h. X-ray photoelectron spectroscopy revealed a strongly fluorinated bond (Y–F bond) on the etched surface of the YF_3_ films. HRTEM analysis also revealed that the YF_3_ films became yttrium-oxyfluorinated after exposure to fluorocarbon plasma. The etching depth was three times lower on YF_3_ film than on Al_2_O_3_ plate. These results showed that the YF_3_ films have excellent erosion resistance properties compared to Al_2_O_3_ plates.

## 1. Introduction

Silicon-oxide series of ceramics, such as SiO_2_ and Al_2_O_3_ coatings, are valued for their hardness, high wear resistance, dielectric strength, high corrosion resistance, and chemical stability. They have been extensively used as plasma-resistant materials in plasma etching equipment and in the deposition thin-film processing semiconductor industry [1,2]. They are also popular shield materials that protect the interior ceramic parts, chamber windows, cover baffles, rings, and reactor chamber walls of plasma equipment from corrosive fluorocarbon gases (e.g., C_2_F_6_, CF_4_, CHF_3_, and C_2_F_6_) [3]. However, on the inner walls of the processing chamber, these oxide materials easily interact with fluorine-based plasma, causing significant erosion and particle generation [4]. As integrated circuits continue to downscale to the nanoscopic level, particle contaminants in their wafers are becoming increasingly worrisome, because they short-current the integration circuit and reduce mass-production yield [5]. Yttrium oxide (Y_2_O_3_) promises to replace SiO_2_ and Al_2_O_3_ as the material of plasma-facing inner walls, owing to its much higher chemical stability and reduced erosion rate [6]_._ Nevertheless, as pointed out by mass-production factories, Y_2_O_3_ inner wall coatings contain pores and crack defects that release particles into the plasma by flake-off [7]. Yttrium fluoride (YF_3_) coatings have recently attracted substantial attention because their high plasma erosion resistance prevents the generation of fluoride particles on the chamber wall surface, reducing particulate contamination [8]. Moreover, YF_3_ reportedly has a high dielectric strength [9]. Thus, YF_3_ coating is a new plasma-facing material. Researchers have fabricated protective coatings in chamber walls using a plasma spray technique. However, as the spray method rapidly forms thick films with porous structures and rough surfaces, it might introduce critical particle impurities during the semiconductor plasma deposition/etching process [10]. Vacuum coating techniques such as magnetron sputtering can address these problems.

To our knowledge, sputtered YF_3_ films and their erosion behavior under fluorocarbon plasma etching had not previously been investigated. Herein, we investigate the composition, structure, erosion behavior, and surface morphology changes of YF_3_ films on sapphire substrate, prepared by magnetron sputtering. 

## 2. Materials and Methods

The sputter material was YF_3_ ceramic target (99.99% purity, 2 inch diameter, 3 mm thickness). YF_3_ thin films were deposited on c-plane sapphire substrate (single crystal of Al_2_O_3_) at room temperature by radio frequency magnetron sputtering in a vacuum chamber. The substrates were cleaned before deposition, first in acetone and alcohol, and then by ultrasonic cleansing in de-ionized (DI) water for 30 min. They were blow-dried in nitrogen gas. The sputtering gas was high-purity argon (99.995%), maintained at a constant flow rate (~100 sccm). The sputtering process was performed under a base chamber pressure of approximately 1.5 × 10^−5^ torr, with turbo molecular and oil diffusion pumps. The plasma generation was activated by RF power at 13.56 MHz. The target–substrate distance was 15 cm. To ensure uniform film thickness, the substrate holder was rotated at 20 rpm during the deposition process. Substrate heating temperature was varied from 400 to 700 °C in steps of 100 °C. The sputter deposition conditions of the YF_3_ films are detailed in Table 1. The plasma etching behaviors of all samples were performed using an inductively-coupled plasma etcher. The etching gases mixed were CF_4_ and O_2_. The etching conditions are shown in Table 2.

The surface morphology, microstructure, and elemental analysis of these coating samples were analyzed by scanning electron microscopy (SEM, S-3000H, Hitachi, Tokyo, Japan) coupled with energy dispersive X-ray diffraction (EDX), atomic force microscopy (AFM, DI-3100, Veeco, New York, NY, USA), and high-resolution transmission electron microcopy (HRTEM, H-600, Hitachi, Tokyo, Japan). The sample compositions were examined by X-ray photoelectron spectroscopy (XPS, PHI 5000 VersaProbe, ULVAC-PHI, Kanagawa, Japan) using a monochromatic Cu Kα X-ray source (*λ* = 1.541874 Å) at a passing energy of 20 eV with a spot size of 650 μm. After XPS, the sample surface was etched using focused argon-ion sputtering to investigate the chemical compositional depth profile (Thermo Scientific K-Alpha). Finally, the photoelectron spectrum resulting from the core energy levels of yttrium 3D states was deconvoluted by a fitting software program (Thermo Fisher Scientific, Inc., Waltham, MA, USA) to estimate the contributions from bonding with fluorine elements. 

## 3. Results and Discussion

**Figure 1:**
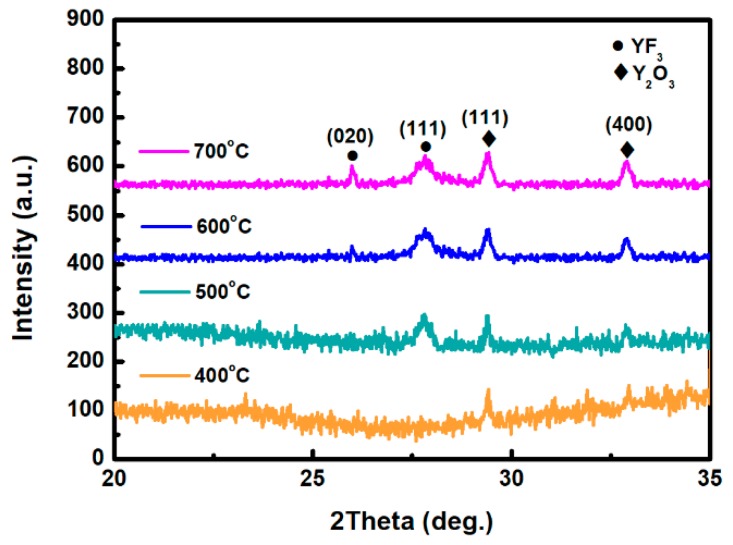
XRD patterns of the as-deposited yttrium fluoride (YF_3_) films grown at 400, 500, 600, and 700 °C.

**Figure 2:**
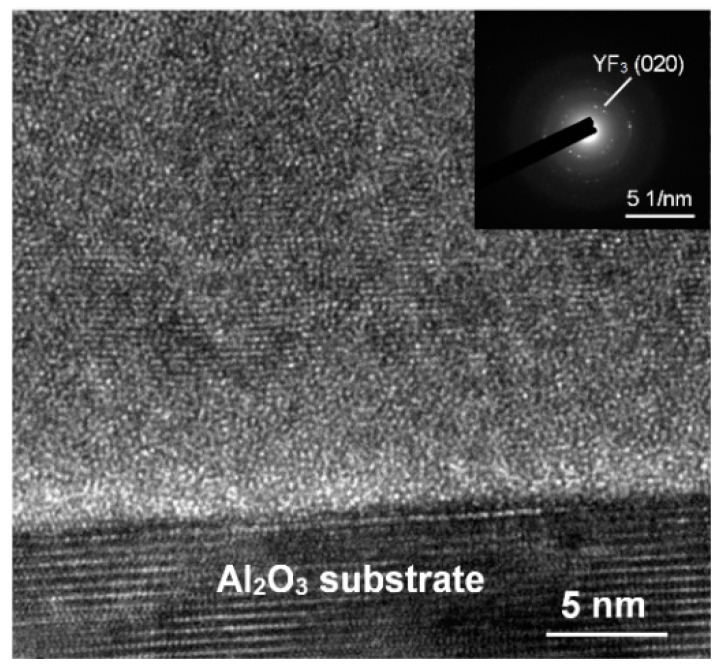
TEM microstructure and selected area diffraction (inset) of YF_3_ film.

Figure 1 depicts the XRD scan patterns of YF_3_ films deposited on Al_2_O_3_ substrate at 400, 500, 600, and 700 °C. All YF_3_ samples were polycrystalline, and their structural orientations depended on the substrate temperature in the deposition process. The (020) plane of the orthorhombic YF_3_ phase was formed at substrate temperatures above 500 °C, which is in agreement with the reported data (JCPDS card files No. 74-0911) [11]. Moreover, the preferential orientation of the orthorhombic YF_3_ crystal structure differed in films fabricated at different temperatures. This might be attributed to the high kinetic energy imparted to YF_3_ molecules at high substrate temperatures, which enables them to migrate and rearrange on the substrate surface. YF_3_ samples fabricated at higher temperatures also showed a weak peak of the cubic Y_2_O_3_ phase (400) plane (JCPDS card files No. 79-1716), indicating contamination by oxygen atoms during the sputtering process [9]. As substrate temperature increases, Y_2_O_3_ formation might be favored by the higher instability of fluoride in the YF_3_ crystal lattice. Unstable F ions in the lattice can be gradually replaced by environmental oxygen atoms. Therefore, the number of oxygen atoms in the crystal lattice increases with increasing temperature, forming Y_2_O_3_ (see Figure 1). When YF_3_ film was deposited at a lower substrate temperature, an amorphous structure was formed. Amorphous film usually has a loose structure, resulting in greater thickness and a higher growth rate. In contrast, with increasing substrate temperature, the YF_3_ film became crystalline, and its crystal was relatively ordered. This indicates that a denser structure was found in the YF_3_ film grown at higher substrate temperatures, leading to its smaller thickness and lower growth rate. In TEM analysis, the YF_3_ films exhibited nanocrystalline grains with an average size of 5–20 nm (Figure 2). The regular ring-like electron diffraction pattern of a selected area (Figure 2, inset) implies a polycrystalline structure of YF_3_ film, consistent with the XRD result.

**Figure 3:**
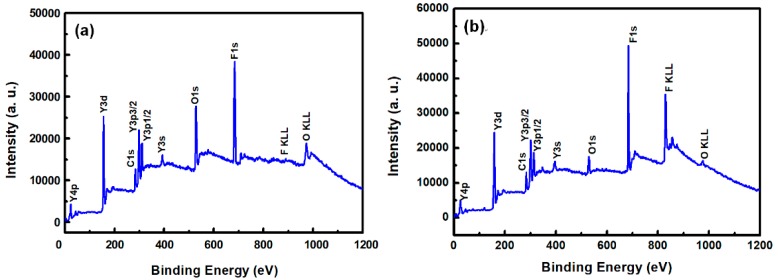
XPS survey spectra of the surfaces of the YF_3_ thin films (**a**) before and (**b**) after exposure to CF_4_/O_2_ plasma (under bombardment with argon ions for two minutes).

**Figure 4:**
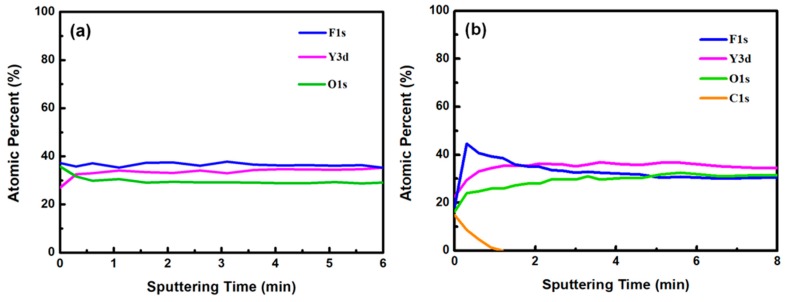
Atomic percentages of YF_3_ film versus sputtering time (**a**) before and (**b**) after surface exposure to CF_4_/O_2_ plasma.

Figure 3a,b show the XPS survey spectra in the 0–1200 eV range of the YF_3_ films before and after CF_4_/O_2_ plasma etching, respectively. To collect the atomic signals, all samples were bombarded by argon ions for 2 min. The XPS peaks in the as-grown YF_3_ thin film (Figure 3a) correspond to Y, F, O, and C elements. The Y and F elements existed in the original YF_3_ film, and an O1s peak arose from oxygen contamination during the sputter deposition process. This result is consistent with previous reports [12]. After exposure to CF_4_/O_2_ plasma, the spectrum of the YF_3_ film exhibited an enhanced F1s peak and an additional C1s peak. The former feature is attributed to the high fluorine content of YF_3_, and the latter indicates the formation of a carbon-polymer surface layer under fluorocarbon plasma etching (Figure 3b). Figure 4a,b plot the compositions of the YF_3_ films as functions of sputtering time before and after exposure to the CF_4_/O_2_ plasma, respectively. Before exposure to the plasma, the maximum F content was 37% and the minimum O content was 32% (Figure 4a). This result indicates a two-phase mixture of YF_3_ and Y_2_O_3_ in the film, consistent with a previous study that reported a deficiency of fluorine atoms during the sputter deposition process [13]. The maximum percentage of F atoms in the etched sample was 44.69%, implying a fluorination layer on the YF_3_ specimen (Figure 4b). Similar results were observed in a previous study of surface fluorination by CF_4_/O_2_ plasma etching [14]. The carbon content on the YF_3_ surface decreased abruptly with sputtering time, indicating a very thin carbon polymer layer on the etched surface. This is because of interactions between the fluorocarbon plasma and Si-based materials, which generate carbon polymer and a SiF_x_O_y_ reaction layer [15].

**Figure 5:**
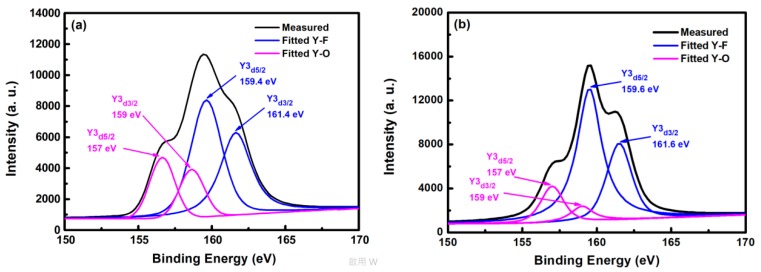
X-ray photoelectron spectra of YF_3_ film (**a**) before and (**b**) after exposure to fluorocarbon plasma.

Figure 5 shows the XPS spectra of yttrium atoms of the YF_3_ film after CF_4_/O_2_ plasma etching. In the curve-fitted XPS spectra of the YF_3_ film, two peaks represent two bonding sources for Y cations, that is, the Y3d peak splits into a doublet (Y3d_5/2_ and Y3d_3/2_ electrons) with a binding-energy separation of 2 eV. The two yttrium bonding sources are consistent with the XPS standard [16]. The Y3d_5/2_ and Y3d_3/2_ peaks in the spectrum of the as-deposited YF_3_ film (Figure 5a) deconvolute into four peaks. The two peaks located at higher binding energies (159.4 and 161.4 eV) correspond to Y–F bonding in the YF_3_ film, whereas those at lower binding energies (157 and 159 eV) are ascribed to Y–O bonding. In the XPS spectra of etched YF_3_ film surface, the peaks are more intense than in the un-etched specimen (Figure 5b). Again, the strong doublet at higher binding energies (159.6 and 161.6 eV) and the weak doublet at lower binding energies (157 and 159 eV) correspond to Y–F and Y–O bonding, respectively. The higher XPS binding energy of the Y–F bonding can be attributed to the higher electronegativity of fluorine atoms (4.0) than oxygen atoms (3.5) [17]. Consequently, more electrons are transferred to fluorine, decreasing the electron density around the cation and enhancing the binding energy. Meanwhile, the intensity ratio of Y–F to Y–O bonds was estimated as 2.1 on etched YF_3_ films, indicating strong chemical interaction between the YF_3_ films and the fluorocarbon plasma.

**Figure 6:**
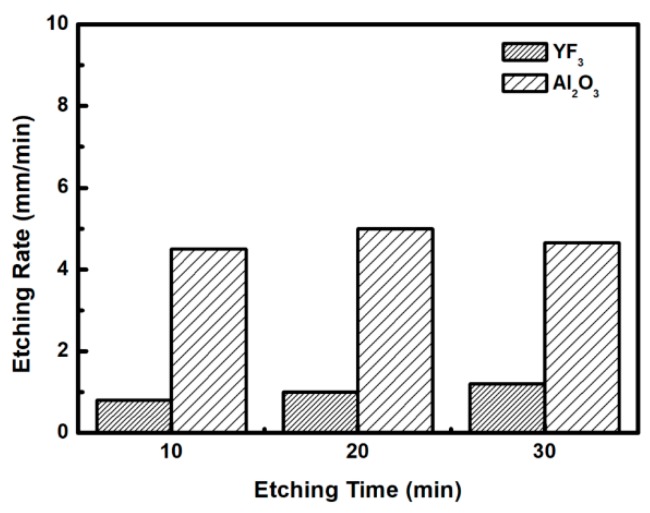
Etching depths of the YF_3_ film and Al_2_O_3_ substrates after different CF_4_/O_2_ plasma exposure times.

**Figure 7:**
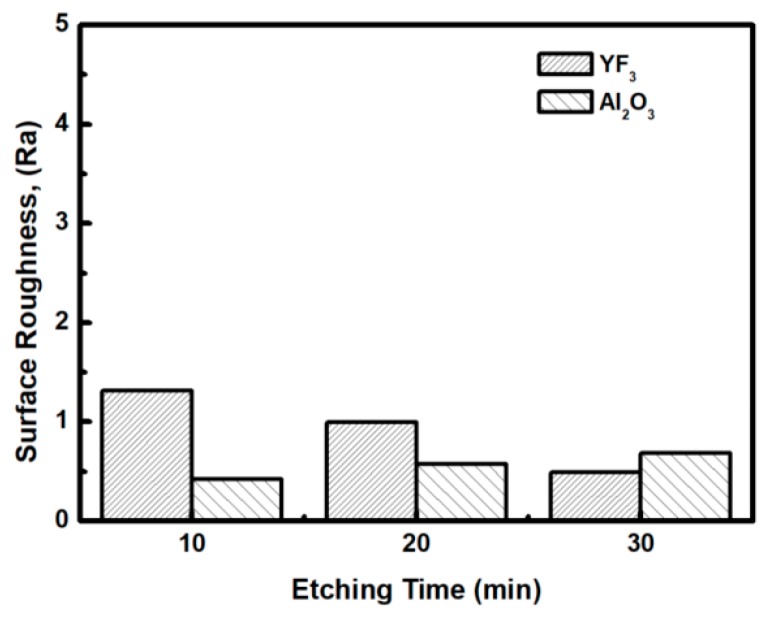
Surface roughness of YF_3_ and Al_2_O_3_ exposed to fluorocarbon plasma for different etching times.

To evaluate the plasma erosion resistance of as-deposited YF_3_ films, the etching rate of the YF_3_-coated Al_2_O_3_ substrate was measured after exposure to the CF_4_/O_2_ plasma for different etching times. The etching rate of bare Al_2_O_3_ crystal was used as the reference. Figure 6 shows that the etching depths of both samples linearly increased with increasing etching time. After 30 min, the etching depth reached 120 mm in the sapphire, but only 38 mm in YF_3_. The erosion resistance of the YF_3_ film to CF_4_/O_2_ plasma etching was more than three times higher than that of sapphire crystal. This is attributed to the chemical stability of YF_3_ in a chemical environment dominated by fluorocarbon plasma. The surface morphologies and roughness of the YF_3_ films etched for different lengths of time were measured by AFM measurements, with a scanning area of 5 m^2^. The surface roughness results are shown in Figure 7. The etching time of the CF_4_/O_2_ plasma did not remarkably influence the surface roughness of the YF_3_ film.

**Figure 8:**
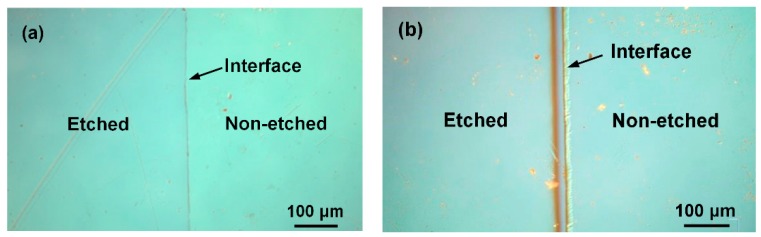
Optical micrographs of the (**a**) YF_3_ and (**b**) Al_2_O_3_ substrates after exposure to fluorocarbon plasma.

Figure 8a,b show the surface microstructures of YF_3_ and Al_2_O_3_ samples, respectively, after the etching experiment. The micrographs were acquired by optical microscopy (OM). The right and left images in each panel show the un-etched and etched surfaces, respectively. The step height of the Al_2_O_3_ sample changed after CF_4_/O_2_ plasma etching. These images confirm the hardness and stronger CF_4_/O_2_ corrosion resistance of the YF_3_ films compared to conventional Al_2_O_3_ substrate.

AlF_3_ is a typical fluoride of Al_2_O_3_ materials [4]. The boiling temperature of YF_3_ (2230 °C) is higher than the sublimation temperature of AlF_3_ (1275 °C). Hence, YF_3_ is more stable and more difficult to vaporize than AlF_3_. Therefore, its fluorinated layer can be removed by a physical sputtering process. The sputtering yields of the films are inversely proportional to sublimation enthalpies of their compounds [18]. The sublimation enthalpy of YF_3_ (481 ± 21 kJ/mol) is higher than that of AlF_3_ (301 ± 4 kJ/mol). Moreover, the enthalpy of formation of the metal–oxygen bond is lower in YF_3_ (−392 kJ mol^−1^) than in Al_2_O_3_ (−279 kJ mol^−1^). Therefore, YF_3_ is chemically more stable than Al_2_O_3_ [19]. In particular, YF_3_ is extremely stable under the fluorocarbon plasma etching process.

**Figure 9:**
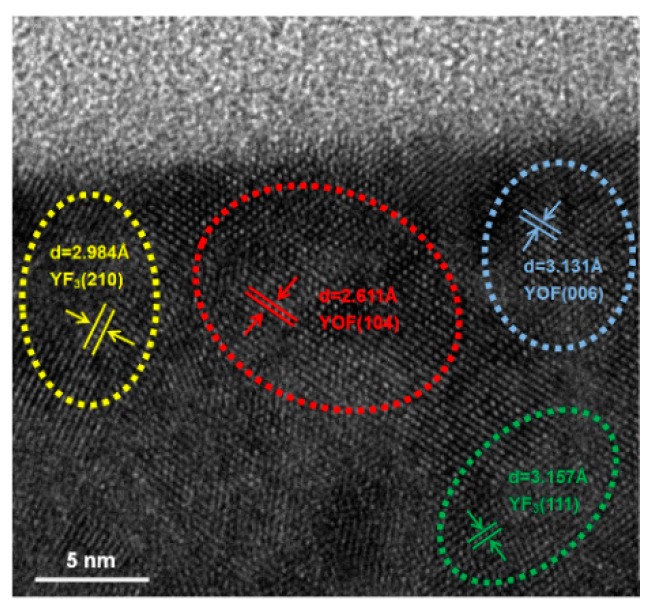
Cross-sectional high-resolution transmission electron microscopy (HRTEM) image of the YF_3_ film after exposure to fluorine plasma.

Figure 9 shows a cross-sectional HRTEM image of the YF_3_ film after CF_4_/O_2_ plasma exposure. Continuous and nearly-complete lattice fringes were observed near the surface, indicating that the YF_3_ ceramic crystalline lattice was not distorted by the fluorine plasma irradiation. Furthermore, the d-spacings of the near surface of film were 3.131 Å (blue dotted circle) and 2.621 Å (red dotted circle), very close to the d-spacings of the altered layers of yttrium oxyflouride (YOF): 3.147 Å (006) and 2.698 Å (104) [11]. The thin YOF layer formed on the YF_3_ surface plays a protective role, suppressing the particle generation during CF_4_/O_2_ plasma etching process. The reaction and formation of an altered YOF layer has been reported previously [14]. Therefore, YF_3_ film is a very attractive plasma-corrosion-resistant material that produces fewer contamination particles during the semiconductor fabrication process.

## 4. Conclusions

YF_3_ films were successfully deposited through radio frequency magnetron sputtering on sapphire substrates at different temperatures. HRTEM and XRD results revealed polycrystalline YF_3_ films with an orthorhombic structure. XPS results confirmed the superior chemical stability of YF_3_ film after fluorocarbon plasma treatment. The robustness of YF_3_ film was confirmed to be more than that of Al_2_O_3_ film after the plasma exposure. The YF_3_ film is expected to provide a more protective barrier than Al_2_O_3_ or quartz plates in the fluorine plasma etching process.

## Figures and Tables

**Table 1:** Experimental parameters of sputter deposition conditions of YF_3_ film.

Parameters	Conditions
RF power (W)	150
Duration (hours)	2
Substrate temperature (°C)	400–700
Substrate rotation (rpm)	20
Working pressure (mTorr)	5
Substrate to target distance (cm)	15
Argon gas flow rate (sccm)	100

**Table 2:** Plasma etching conditions.

Parameters	Condition
RF power (W)	800
RF power, bias (W)	100
Pressure (mTorr)	3
CF_4_ (sccm)	25
O_2_ (sccm)	5
Etching time (min)	10, 20, 30
